# Risk factors and impact of early anastomotic biliary complications after liver transplantation: UK registry analysis

**DOI:** 10.1093/bjsopen/zrab019

**Published:** 2021-04-15

**Authors:** S J Tingle, E R Thompson, S S Ali, R Figueiredo, M Hudson, G Sen, S A White, D M Manas, C H Wilson

**Affiliations:** 1 National Institute for Health Research Blood and Transplant Research Unit (NIHR BTRU) in Organ Donation and Transplantation, Institute of Transplantation, Freeman Hospital, Newcastle upon Tyne UK; 2 Faculty of Medical Sciences, Imperial College London, South Kensington, London, UK; 3 Department of Hepatology, Freeman Hospital, Newcastle upon Tyne, UK

## Abstract

**Background:**

Biliary leaks and anastomotic strictures are common early anastomotic biliary complications (EABCs) following liver transplantation. However, there are no large multicentre studies investigating their clinical impact or risk factors. This study aimed to define the incidence, risk factors and impact of EABC.

**Methods:**

The NHS registry on adult liver transplantation between 2006 and 2017 was reviewed retrospectively. Adjusted regression models were used to assess predictors of EABC, and their impact on outcomes.

**Results:**

Analyses included 8304 liver transplant recipients. Patients with EABC (9·6 per cent) had prolonged hospitalization (23 *versus* 15 days; *P* < 0·001) and increased chance for readmission within the first year (56 *versus* 32 per cent; *P* < 0·001). Patients with EABC had decreased estimated 5-year graft survival of 75·1 *versus* 84·5 per cent in those without EABC, and decreased 5-year patient survival of 76·9 *versus* 83·3 per cent; both *P* < 0.001. Adjusted Cox regression revealed that EABCs have a significant and independent impact on graft survival (leak hazard ratio (HR) 1·344, *P* = 0·015; stricture HR 1·513, *P* = 0·002; leak plus stricture HR 1·526, *P* = 0·036) and patient survival (leak HR 1·215, *P* = 0·136, stricture HR 1·526, *P* = 0·001; leak plus stricture HR 1·509; *P* = 0·043). On adjusted logistic regression, risk factors for EABC included donation after circulatory death grafts, graft aberrant arterial anatomy, biliary anastomosis type, vascular anastomosis time and recipient model of end-stage liver disease.

**Conclusion:**

EABCs prolong hospital stay, increase readmission rates and are independent risk factors for graft loss and increased mortality. This study has identified factors that increase the likelihood of EABC occurrence; research into interventions to prevent EABCs in these at-risk groups is vital to improve liver transplantation outcomes.

## Teaser

Early anastomotic biliary complications are common following liver transplantation. In this national registry analysis we show that these complications have a significant, independent, impact on graft loss and mortality following transplantation. We also identify a number of key risk factors for development of early anastomotic biliary complications.

## Introduction

Liver transplantation is the definitive treatment option for patients with end-stage liver failure with average 5-year survival rates around 80 per cent in the UK^1^. The success of liver transplantation has led to increasing demand for organs. However, the supply of high-quality grafts is limited. This has led to the increased use of marginal grafts from extended-criteria donors and donation following circulatory death (DCD) donors^2–4^. These organs pose an increased risk of primary non-function and higher rates of both vascular and biliary anastomotic complications^4–6^.

Biliary complications are the most commonly reported complications following liver transplantation^7^. Incidence has been estimated to be approximately 10 to 15 per cent in deceased donor transplants^8^ and may be as high as 15 to 30 per cent in living donor transplant recipients^9^. These complications are often associated with split grafts, concomitant vascular complications, prolonged ischaemia, reperfusion injury, cytomegalovirus (CMV) infection, primary sclerosing cholangitis (PSC) and the type of biliary reconstruction^10–14^. More than half of all biliary complications are early complications occurring at the anastomotic site. Approximately 30 per cent represent early anastomotic strictures and around 20 per cent are bile leaks^7^. These early anastomotic biliary complications (EABCs) are the focus of this study.

There are a number of biliary reconstruction options for surgeons including end-to-end choledocho-choledochostomy (CDCD) anastomosis, Roux-en-Y hepaticojejunostomy, T-tube, or stented anastomosis. Currently, end-to-end ductal anastomosis is the most commonly used biliary reconstruction in liver transplantation, however the impact of anastomosis technique on biliary complication rates remains poorly described.

A number of single-centre studies have previously investigated the impact of EABC on morbidity and mortality^15–17^. However, these are underpowered to investigate the impact of EABC on long-term graft survival and mortality. Furthermore, small studies are unable to adjust for sufficient confounders, and therefore cannot evaluate whether EABCs are independent predictors of poor outcome, or simply markers of poor grafts and complex transplants. Whereas registry analyses have been performed to investigate economic implications, there have been, to the authors’ knowledge, no large multicentre analyses investigating the clinical impact of early biliary complications^18^.

There is a significant volume of research examining the best way to manage biliary complications after transplantation^19–21^ but robust studies describing how best to prevent them are lacking. Many clinicians feel the development of a biliary complication is a multifactorial problem and may be indicative of a poor graft or poor preservation. However, a formal analysis of risk factors for EABC development using a sufficiently large database has not been performed. As increasingly marginal organs are transplanted it is important to identify risk factors that are amenable to intervention.

The aim of this study is to investigate the incidence, impact and risk factors associated with EABC with a view to better informing surgeons which donor/recipient combinations and operative factors may increase the risk of developing this common complication.

## Methods

National UK data on adult liver transplantation from eight transplant centres between 2006 and 2017 collected and validated by the National Health Service Blood and Transplant (NHSBT) transplant registry were reviewed. Data were provided in an anonymized form (patient-identifiable information and transplant unit not provided) as per NHSBT approvals, and individual ethical approval was not required for this project. All adult liver transplants were included. The NHSBT registry records occurrence of surgical complications within 3 months as a dichotomous variable. EABCs were therefore recorded as the development of a leak and/or stricture within the first 3 months after transplantation. For analysis the cohort was split into four groups; no EABC, leak only, stricture only and leak plus stricture.

Data was cleaned, and values which were deemed impossible were removed (summary of removed data points is in *Table S1*). Of this final cohort, 68 patients were noted as either to have no biliary stricture (67 patients) or not reported (1 patient), despite having a form of biliary stricture treatment. The authors assumed this was a mistake in the data and characterized these participants as having a stricture. Sensitivity analysis was performed where graft survival, patient survival and predictors were re-analysed with these 68 mismatches excluded, or included in the no stricture groups; the results did not change significantly.

### Statistical analysis

All patients included had a minimum of 12 months’ follow-up. Initial crude analyses compared donor, recipient, operative and early outcomes in the four biliary complication groups. Pearson’s χ^2^ test was used for categorical variables. If cell counts were too low, then Monte Carlo estimates of Fisher’s exact tests were used. For continuous data, normality was evaluated using Kolmogorov–Smirnov tests. Analysis of variance (ANOVA) and Kruskal–Wallis tests were then applied as appropriate. Data results are mean(s.d.) or median (range), depending on normality. Post-hoc tests with Bonferroni correction were applied. All tests were two-sided. The common closure date of the study was June 2018; graft and patient survival were censored at 10 years. Unadjusted graft and patient survival were calculated using Kaplan–Meier plots and *P* values were derived with the log-rank test. Graft survival was death censored, and graft loss was defined as retransplant or death due to liver failure.

A rigorous approach was taken to build the adjusted multivariable Cox regression models for graft and patient survival. Donor, graft, recipient, operative and postoperative factors available from NHSBT registry were initially screened, and a broad selection criterion was applied. Variables were selected if they had previously been reported to affect graft or patient survival^22^^,^^23^, were significantly correlated with EABCs, or were associated with survival on univariable Cox regression in the study cohort (P < 0·200). Multiple imputation was used to generate 20 imputed datasets, as described below. Cox regression with backward likelihood ratio stepwise selection was then performed to identify key variables. Variables retained in more than 90 per cent of imputed datasets were included in a final adjusted multivariable model using pooled data from all 20 imputations. Sensitivity analyses were performed which included variables retained in more than 50 per cent of imputed models, and none of the extra variables was significant in the final models.

Multiple binary logistic regression was used to identify significant predictors of biliary leak and biliary stricture. A model was built using an identical approach to that described above. Results displayed are adjusted hazard ratios (aHR) or adjusted odds ratios (aOR) with 95 per cent confidence intervals.

Variables with the largest proportion of missing data were donor bilirubin (29·0 per cent), recipient diabetes (23·2 per cent), single *versus* multiple hepatic artery anastomosis (18·0 per cent), graft hepatic artery anatomy (16·6 per cent), vascular anastomosis time (16·1 per cent), waiting list time (14·0 per cent), UK model for end-stage liver (UKELD, 13.6 per cent), model for end-stage liver disease (MELD, 13·6 per cent), donor cardiovascular disease (5·6 per cent) and donor hypertension (5·2 per cent). All remaining variables had missing data in less than 5 per cent of cases (full report of missing data in *Table S2*). Missing data were dealt with by multiple imputation using the fully conditional specification technique applied to generate 20 imputed datasets.

For all tests performed *P* < 0·050 was deemed significant. All analyses were performed in SPSS^TM^ version 26 (IBM Corp, Armonk, New York, USA), and figures were generated using GraphPad Prism^TM^ version 6 (GraphPad Software, San Diego, California USA).

## Results

The initial database included 8780 adult liver transplant recipients. The following patients were excluded: multivisceral transplants (148 patients), missing data on leak and stricture (198 patients), missing graft survival data (30 patients), intraoperative deaths (35 patients), or graft loss on day 0 or 1 (65 patients). The last two groups were excluded to minimize immortal time bias, as leak or stricture would never be recorded. This left 8304 participants for the main analyses. *Table 1* summarizes the demographics of this cohort.

**Table 1:** Demographic information for the entire dataset

	**Cohort demographics** **(*n* = 8304)**

**Donor characteristics**	
Donor age (years)^†^	50.0 (6–86)
Cold ischaemic time (min)^*^	511.8 (163.5)
Donor type	
DBD	6476 (78.0)
DCD	1677 (20.2)
Living	111 (1.3)
Domino	40 (0.5)
**Recipient and transplant characteristics**	
Recipient age (years)^†^	54.0 (18–75)
Recipient MELD^*^	16.6 (6.84)
Biliary anastomosis type	
CDCD	6599 (80.2)
Roux-en-Y	1261 (15.3)
T-tube	330 (4.0)
Stent	40 (0.5)
**Early anastomotic biliary complication**	
No biliary complication	7505 (90.4)
Biliary leak only	344 (4·14)
Biliary stricture only	335 (4·03)
Both leak and stricture	120 (1·44)

Values in parentheses are percentages unless indicated otherwise;

* values are mean(s.d.);

† values are median (range). DBD, donation following brainstem death; DCD, donation following circulatory death; MELD, model for end-stage liver disease; CDCD, choledocho–choledochostomy.

Overall, EABCs were reported in 799 (9·6 per cent) patients: 344 (4·14 per cent) experienced a biliary leak, 335 (4·03 per cent) a biliary stricture and 120 (1·44 per cent) had both leak and stricture.

### Clinical demographics

The cohort was split into these four groups: those with no biliary complication, leak only, stricture only and a combination of leak and stricture. These groups were well matched for most clinical demographics (*Tables 2–4*).

**Table 2:** Donor factors associated with early anastomotic biliary complications

Donor factor	**No biliary complication** **(*n* = 7505)**	**Biliary leak alone** **(*n* = 344)**	**Biliary stricture alone** **(*n* = 335)**	**Both leak and stricture** **(*n* = 120)**	*P* ^‡^
Donor age*	50 (6–86) (*n* = 7504)	45 (6–83)	48 (11–85)	49.5 (18–79)	0·001^¶^
**Donor sex**					
Male	3927 (90·2)	174 (4·0)	189 (4·3)	65 (1·5)	0·428
Female	3575 (90·6)	170 (4·3)	146 (3·7)	55 (1·4)	
**Cold ischaemic time (min)** ^†^	512(162) (*n* = 7436)	522(183), (*n* = 336)	498(169), (*n* = 329)	509(189), (*n* = 117)	0·298^¶^
**Total warm ischaemic time in DCD grafts (min)** ^†^	27·0(8·2) (*n* = 818)	29·0(10·7) (*n* = 33)	27·6(7·1) (*n* = 50)	27·9(8·8) (*n* = 18)	0·536^¶^
**Donor type**					
DBD	5887 (90·9)	264 (4·1)	245 (3·8)	80 (1·2)	<0·001^§^
DCD	1505 (89·7)	61 (3·6)	82 (4·9)	29 (1·7)	
Living	78 (70·3)	15 (13·5)	7 (6·3)	11 (9·9)	
Domino	35 (87·5)	4 (10·0)	1 (2·5)	0 (0)	
**Steatosis**					
No steatosis	4087 (90·4)	196 (4·3)	181 (4·0)	57 (1·3)	0·709
Mild	2302 (90·7)	98 (3·9)	104 (4·1)	35 (1·4)	
Moderate	835 (91·4)	29 (3·2)	36 (3·9)	14 (1·5)	
Severe	72 (93·5)	1 (1·3)	4 (5·2)	0 (0)	
**Transplant type**					
Whole liver	6926 (91·2)	267 (3·5)	307 (4·0)	95 (1·3)	<0·001
Reduced or split liver	579 (81·7)	77 (10·9)	28 (3·9)	25 (3·5)	
**Ethnicity**					
White	7028 (90·6)	309 (4·0)	308 (4·0)	109 (1·4)	0·008^§^
Asian	160 (89·9)	8 (4·5)	8 (4·5)	2 (1·1)	
Black	106 (91·4)	4 (3·4)	4 (3·4)	2 (1·7)	
Other	150 (80·2)	18 (9·6)	14 (7·5)	5 (2·7)	
**Donor BMI** ^†^	26·1(4·8) (*n* = 7395)	25·5(4·7) (*n* = 328)	26·2(5·1) (*n* = 327)	26·6(4·8) (*n* = 109)	0·076^¶^
**Donor past medical history**					
Diabetic	426 (91·2)	7 (1·5)	28 (6·0)	6 (1·3)	0·005
Non-diabetic	6777 (90·6)	311 (4·2)	290 (3·9)	99 (1·3)	
Hypertensive	1916 (91·1)	67 (3·2)	98 (4·7)	23 (1·1)	0·027
Non-hypertensive	5221 (90·5)	249 (4·3)	217 (3·8)	81 (1·4)	
History of alcohol abuse	607 (90·7)	23 (3·4)	30 (4·5)	9 (1·3)	0·669
No alcohol abuse	4927 (90·8)	214 (3·9)	200 (3·7)	84 (1·5)	
CVS disease	712 (91·6)	24 (3·1)	30 (3·9)	11 (1·4)	0·549
No CVS disease	6392 (90·6)	293 (4·2)	280 (4·0)	93 (1·3)	
History of liver disease	82 (83·7)	8 (8·2)	6 (6·1)	2 (2·0)	0·064^§^
No liver disease	7036 (90·8)	305 (3·9)	310 (4·0)	102 (1·3)	
Smoker	3715 (90·9)	169 (4·1)	153 (3·7)	49 (1·2)	0·409
Non-smoker	3515 (90·6)	145 (3·7)	164 (4·2)	56 (1·4)	

Values in parentheses are percentages unless otherwise indicated. Percentages displayed describe biliary complication rate for each donor factor (within-row percentages).

* Values are median (range);

† values are mean(s.d.).

‡ χ^2^ test, except

§ Monte Carlo simulations of Fishers exact test,

¶ one-way ANOVA. Raw data are given, a summary of missing data is found in *Table S2*. Further donor factors are displayed in *Table S3*.

**Table 3:** Recipient factors associated with early anastomotic biliary complications

Recipient factor	No biliary complication (*n* = 7505)	**Biliary leak alone** **(*n* = 344)**	**Biliary stricture alone** **(*n* = 335)**	**Both leak and stricture** **(*n* = 120)**	*P* ^‡^
Recipient age*	54 (17–75)	54 (17–73)	53 (19–73)	55 (20–72)	0·224
**Recipient sex**					
Male	4675 (90·8)	196 (3·8)	197 (3·8)	83 (1·6)	0·045
Female	2829 (89·8)	148 (4·7)	138 (4·4)	37 (1·2)	
**Indication**					
Non-biliary	6185 (90·5)	267 (3·9)	285 (4·2)	97 (1·4)	0·013^§^
PBC	600 (88·2)	36 (5·3)	35 (5·1)	9 (1·3)	
PSC	692 (91·4)	39 (5·2)	14 (1·8)	12 (1·6)	
Biliary atresia	28 (84·8)	2 (6·1)	1 (3·0)	2 (6·1)	
**Listed as super-urgent**	910 (91·1)	37 (3·7)	42 (4·2)	10 (1·0)	0·527
Not listed as super-urgent	6595 (90·3)	307 (4·2)	293 (4·0)	110 (1·5)	
**Liver failure grade**					
Not acute	6335 (90·4)	288 (4·1)	275 (3·9)	106 (1·5)	0·022
Hyperacute	618 (92·9)	22 (3·3)	18 (2·7)	7 (1·1)	
Acute	224 (89·2)	8 (3·2)	17 (6·8)	2 (0·8)	
Subacute	148 (87·1)	10 (5·9)	12 (7·1)	0	
**MELD**†	16·6(6·8) (*n* = 6493)	17·7(7·5) (*n* = 295)	16·2(6·8) (*n* = 285)	16·5(7·3) (*n* = 104)	0·048^¶^

Values in parentheses are percentages unless indicated otherwise. Percentages displayed describe biliary complication rate for each recipient factor (within-row percentages).

* Values are median (range);

† values are mean(s.d.).

‡ χ^2^ test, except

§ Monte Carlo simulations of Fishers exact test,

¶ one-way ANOVA. Raw data are given, a summary of missing data is found in *Table S2*. Further recipient factors are displayed in *Table S3*. PBC, primary biliary cirrhosis; PSC, primary sclerosing cholangitis; MELD, model for end-stage liver disease.

**Table 4:** Operative factors associated with early anastomotic biliary complications

Operative factor	**No biliary complication** **(*n* = 7505)**	**Biliary leak alone** **(*n* = 344)**	**Biliary stricture alone** **(*n* = 335)**	**Both leak and stricture** **(*n* = 120)**	***P*** ^†^
**Transplant year**					
2006–2009	2034 (91·3)	99 (4·4)	67 (3·0)	27 (1·2)	0·086
2010–2013	2357 (89·9)	103 (3·9)	119 (4·5)	44 (1·7)	
2014–2017	3114 (90·2)	142 (4·1)	149 (4·3)	49 (1·4)	
**Biliary anastomosis**					
CDCD	5967 (90·4)	235 (3·6)	291 (4·4)	106 (1·6)	<0·001**^‡^**
Roux	1153 (91·4)	70 (5·6)	27 (2·1)	11 (0·9)	
T-tube	282 (85·5)	34 (10·3)	12 (3·6)	2 (0·6)	
Stent	35 (87·5)	4 (10)	1 (2·5)	0	
**Donor HA anatomy**					
Single	4997 (90·2)	218 (3·9)	238 (4·3)	87 (1·6)	0·114
Accessory	1222 (88·2)	71 (5·1)	71 (5·1)	22 (1·6)	
**HA anastomosis**					
Single	5255 (89·9)	238 (4·1)	258 (4·4)	93 (1·6)	0·831
Multiple	860 (89·1)	45 (4·7)	45 (4·7)	15 (1·6)	
Vascular anastomosis time (mins)*	39 (10–217) (*n* = 6258)	41 (17–181) (*n* = 276)	38 (13–95) (*n* = 290)	42 (13–102) (*n* = 109)	0·002^§^

Values in parentheses are percentages unless indicated otherwise. Percentages displayed describe biliary complication rate for each operative factor (within-row percentages).

* Values are median (range).

† χ^2^ test, except

‡ Monte Carlo simulations of Fishers exact test,

§ Kruskall–Wallis test. Raw data are given, a summary of missing data is found in *Table S2*. CDCD, choledocho-choledochostomy, Roux, Roux-en-Y hepaticojejunostomy; HA, hepatic artery.

Donor demographics are described in *Table 2*. Donor age differed between groups on unadjusted analysis, with post-hoc tests revealing significantly lower age in the biliary leak group compared with no biliary complication. Grafts from DCD, living donors and domino all had higher rates of EABC, as did split or reduced grafts, grafts from diabetic donors and grafts from donors with ethnicity ‘other’.

*Table 3* describes recipient demographics between groups. Female recipients or those with primary biliary cirrhosis or biliary atresia experienced more biliary complications, whilst those patients transplanted for acute liver failure had fewer EABCs. Post-hoc tests revealed that those in the leak alone group had significantly higher MELD than those with no EABCs.

Perioperative factors are shown in *Table 4*. The type of biliary anastomosis was associated with differing rates of EABC. On post-hoc tests, vascular anastomosis time (also termed second warm ischaemic time) was higher in the leak and leak plus stricture groups than in the no EABC group. Association of additional variables stored in the registry with EABCs can be found in *Table S3*. All variables in any of these tables displaying significant differences across the four groups were selected as potential variables for inclusion into Cox regression models.

### Association with other early postoperative complications

There is a strong association between EABCs and most other early complications recorded by NHSBT (*Table 5*). EABC rates were significantly higher in patients experiencing any of the following early complications: acute rejection, renal failure requiring dialysis, hepatic artery thrombus, active CMV infection, fungal infection, early sepsis. Data on these other complications are collected at the same time as EABC, and the database does not contain information on exactly when, or in which order, these complications develop. All of these early postoperative complications were therefore included as potential confounders in the adjusted models.

**Table 5:** Early postoperative complications (within 3 months) associated with early anastomotic biliary complications

Postoperative factor	**No biliary complication** **(*n* = 7505)**	**Biliary leak alone** **(*n* = 344)**	**Biliary stricture alone** **(*n* = 335)**	**Both leak and stricture** **(*n* = 120)**	P*
**Acute rejection episodes**					
0	6160 (91·1)	257 (3·8)	255 (3·8)	91 (1·3)	<0·001
1	1055 (89·0)	63 (5·3)	48 (4·0)	20 (1·7)	
≥2	212 (80·6)	21 (8·0)	25 (9·5)	5 (1·9)	
**Postoperative RRT**					
Nil	5956 (90·7)	256 (3·9)	256 (3·9)	100 (1·5)	0·002^†^
Transient filtration	870 (90·4)	46 (4·8)	37 (3·8)	9 (0·9)	
Short-term dialysis	571 (88·4)	31 (4·8)	34 (5·3)	10 (1·5)	
Long-term dialysis	21 (67·7)	6 (19·4)	3 (9·7)	1 (3·2)	
**HA thrombus**	233 (82·9)	30 (10·7)	11 (3·9)	7 (2·5)	<0·001
No HA thrombus	7272 (90·7)	314 (3·9)	324 (4·0)	111 (1·4)	
**PV thrombus**	227 (87·6)	13 (5·0)	13 (5·0)	6 (2·3)	0·410
No PV thrombus	7278 (90·5)	330 (4·1)	322 (4·0)	112 (1·4)	
**CMV infection**	583 (82·7)	63 (8·9)	51 (7·2)	8 (1·1)	<0·001
No CMV infection	6900 (91·1)	280 (3·7)	284 (3·7)	111 (1·5)	
**Fungal infection**	147 (79·9)	25 (13·6)	8 (4·3)	4 (2·2)	<0·001
No fungal infection	7335 (90·6)	317 (3·9)	326 (4·0)	116 (1·4)	
**Early sepsis**	2467 (84·9)	217 (7·5)	173 (6·0)	50 (1·7)	<0·001
No sepsis	4997 (93·4)	127 (2·4)	160 (3·0)	68 (1·3)	

Values in parentheses are percentages. Percentages displayed describe biliary complication rate for each postoperative factor (within-row percentages).

* χ^2^ test, except

† Monte Carlo simulations of Fishers exact test. Raw data are given, a summary of missing data is found in *Table S2*. RRT, renal replacement therapy; HA, hepatic artery; PV, portal vein; CMV, cytomegalovirus.

### Association with 1-year outcomes

Association between occurrence of EABC and outcomes at 1 year is displayed in *Table 6*. Patients experiencing any EABC spent approximately 50 per cent longer in hospital on their initial admission when compared with the rest of the cohort (23 *versus* 15 days; *P* < 0·001). They were also over 75 per cent more likely to need readmission to hospital in the first year after transplantation (56 per cent *versus* 32 per cent; *P* < 0·001). Recipients with a biliary stricture were significantly more likely to have ongoing renal dysfunction 1 year after transplant compared with those with no biliary complication.

**Table 6:** Outcomes at 1-year separated by biliary complication

Outcomes at 1-year	**No biliary complication** **(n = 7505)**	**Biliary leak alone** **(n = 344)**	**Biliary stricture alone** **(n = 335)**	**Both leak and stricture** **(n = 120)**	***P*** ^†^
Duration of hospital stay in days (index admission) *	15 (4–378) (*n* = 7020)	26 (7–119) (*n* = 303)	20·5 (6–108) (*n* = 310)	26 (8–402) (*n* = 113)	<0·001**^‡^**
**Readmissions (1-year)**					
0	4195 (67·6)	127 (46·5)	114 (42·5)	41 (40·2)	<0·001
1	1143 (18·4)	70 (25·6)	67 (25·0)	22 (21·6)	
>1	870 (14)	76 (27·8)	87 (32·5)	39 (38·2)	
**Renal dysfunction (1-year)**	1393 (22·8)	55 (20·3)	75 (28·4)	16 (16)	0·040
No renal dysfunction	4729 (77·2)	216 (79·7)	189 (71·6)	84 (84)	
**1-year blood results**					
Bilirubin (μmol/l)^*^	10 (1–977) (*n* = 6183)	10 (3–790) (*n* = 268)	10 (2–676) (*n* = 267)	10 (3–455) (*n* = 102)	0·344**^‡^**
ALP (unit/l)^*^	126 (10–1995) (*n* = 6146)	140·5 (11–1917) (*n* = 266)	168·5 (44–1306) (*n* = 266)	205 (9–1749) (*n* = 99)	<0·001**^‡^**
ALT (unit/l)^*^	26 (3–1371) (*n* = 3613)	28 (6–1238) (*n* = 174)	27 (6–332) (*n* = 178)	37·5 (8–274) (*n* = 92)	0·045**^‡^**
AST (unit/l)^*^	23 (2–1814) (*n* = 3565)	27 (6–780) (*n* = 152)	27 (8–2219) (*n* = 149)	38 (13–179) (*n* = 31)	0·001**^‡^**

Values in parentheses are percentages except where indicated otherwise. Percentages represent the chances of renal dysfunction or readmission in each of the biliary complication groups (within column percentage).

* Values are median (range).

† χ^2^ test, except

‡ Kruskall-Wallis test. ALP, alkaline phosphatase; ALT, alanine transaminase; AST, aspartate aminotransferase.

Liver function tests at 1 year were also deranged in the EABC groups (*Table 6*). On post-hoc tests, alkaline phosphatase was significantly higher in all three EABC groups when compared with the no EABC group. Median aspartate aminotransferase and alanine transaminase were significantly different between groups, and numerically higher in the EABC groups. However, none of the comparisons reached statistical significance on post-hoc tests.

### Graft survival

**Fig. 1:**
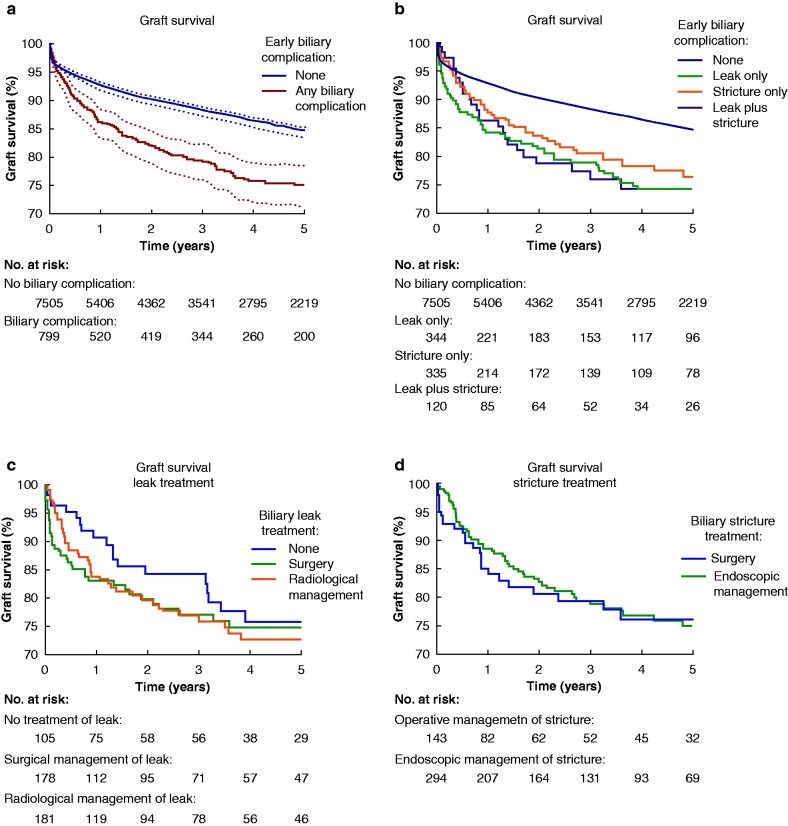
Impact of early anastomotic biliary complications on graft survival Each panel is a Kaplan–Meier graph, with numbers at risk displayed. *P* values are the result of log-rank tests. **a** *P* < 0.001. Shaded areas represent 95 per cent confidence intervals. **b** *P* < 0.001. **c** *P* = 0.883. **d** *P* = 0.683

*Figure 1* displays unadjusted analysis of graft survival in Kaplan-Meier plots. Patients experiencing EABC had significantly worse graft survival (*Fig. 1a,b*). Overall, estimated 5-year graft survival was 84·5 (95 per cent c.i. 83·5–85·5) per cent in patients without an EABC, and 75·1 (95 per cent c.i. 71·3 to 78·5) per cent in the any biliary complication group; *P* < 0·001(*Fig. 1a*). In the case of leak, the authors did not find a significant difference in graft survival for patients managed conservatively, radiologically or surgically (*Fig. 1c*). The vast majority (437 of 455; 96 per cent) of patients with a stricture received treatment. There were no graft-survival differences seen between endoscopic and surgical management of stricture (*Fig. 1d*). However, these are unadjusted analyses and selection bias will exist.

In the final adjusted multivariable Cox regression model (*Table 7*) early anastomotic biliary complications were significant independent risk factors for graft loss (leak only aHR 1·344 (95 per cent c.i. 1·059 to 1·705), *P* = 0·015; stricture only aHR 1·513 (1·171 to 1·956), *P* = 0·002; leak plus stricture aHR 1·526 (1·028 to 2·264), *P* = 0·036). Early biliary complications were retained as significant predictors of graft survival in all 20 of the imputed datasets.

**Table 7:** Graft survival Cox regression using pooled data from all 20 imputed datasets (*n* = 8304 per imputation).

	Univariable		Adjusted	
Variable	Hazard ratio	*P*	**Hazard** **ratio**	*P*
;**Biliary complication**				
None	1	–	1	–
Leak only	1·761 (1·398–2·217)	<0·001	1·344 (1·059–1·705)	0·015
Stricture only	1·571 (1·220–2·023)	<0·001	1·513 (1·171–1·956)	0·002
Leak plus stricture	1·726 (1·169–2·547)	0·006	1·526 (1·028–2·264)	0·036
**Indication**				
Non-biliary	1	–	1	–
Primary biliary cirrhosis	0·796 (0·633–1·002)	0·052	0·868 (0·687–1·098)	0·238
Primary sclerosing cholangitis	1·517 (1·277–1·802)	<0·001	1·624 (1·356–1·943)	<0·001
Biliary atresia	1·918 (0·956–3·847)	0·067	2·126 (1·050–4·302)	0·036
**Liver failure grade**				
Not acute	1		1	
Hyperacute	0·905 (0·730–1·121)	0·362	0·710 (0·549–0·917)	0·009
Acute	1·241 (0·923–1·669)	0·152	1·310 (0·958–1·793)	0·091
Subacute	0·653 (0·405–1·055)	0·082	0·751 (0·461–1·223)	0·250
**Transplant year**	0·987 (0·968–1·006)	0·190	0·960 (0·941–0·980)	<0·001
**Type of transplant**				
Whole	1		1	
Reduced	0·789 (0·457–1·364)	0·397	0·801 (0·199–3·229)	0·755
Split	1·149 (0·936–1·411)	0·184	1·642 (1·299–2·075)	<0·001
**Type of graft**				
DBD	1		1	
DCD	1·460 (1·273–1·675)	<0·001	1·818 (1·557–2·122)	<0·001
Living	0·963 (0·531–1·746)	0·902	1·559 (0·337–7·216)	0·570
Domino	1·793 (0·930–3·458)	0·081	2·914 (1·497–5·673)	0·002
**Previous non-liver transplants**				
0	1		1	
1	3·173 (1·702–5·913)	<0·001	4·113 (2·047–8·262)	<0·001
2	4·783 (0·673–33·992)	0·110	6·723 (0·936–48·306)	0·058
**Previous liver transplants**				
0	1		1	
1	1·648 (1·379–1·970)	<0·001	1·802 (1·485–2·100)	<0·001
>1	2·616 (1·785–3·834)	<0·001	3·753 (2·540–5·545)	<0·001
**Donor age**	1·007 (1·003–1·010)	<0·001	1·013 (1·009–1·017)	<0·001
**Donor diabetes**	1·361 (1·088–1·703)	0·007	1·480 (1·181–1·855)	0·001
**Donor smoker**	1·147 (1·021–1·287)	0·021	1·196 (1·061–1·348)	0·003
**CIT (per 10 mins)**	1·004 (1·000–1·007)	0·041	1·005 (1·001–1·009)	0·015
**Acute rejection episodes**				
0	1		1	
1	1·123 (0·961–1·311)	0·145	1·189 (1·014–1·394)	0·033
>1	1·379 (1·051–1·809)	0·020	1·381 (1·049–1·818)	0·021
**Postoperative RRT**				
Nil	1		1	
Transient filtration	1·802 (1·536–2·113)	<0·001	1·907 (1·599–2·273)	<0·001
Short-term dialysis	2·448 (2·069–2·898)	<0·001	2·430 (2·039–2·896)	<0·001
Long-term dialysis	4·460 (2·473–8·043)	<0·001	3·551 (1·915–6·585)	<0·001
**HA thrombus**	12·212 (10·433–14·296)	<0·001	10·749 (9·122–12·665)	<0·001
**Fungal infection**	2·025 (1·493–2·748)	<0·001	1·484 (1·086–2·029)	0·013

Values in parentheses are 95 per cent confidence intervals. These variables were selected using a backwards stepwise approach. Interaction term between previous liver and non-liver transplants included in model but not displayed. DBD, donation following brainstem death; DCD, donation following circulatory death; CIT, cold ischaemic time, RRT, renal replacement therapy; HA, hepatic artery.

When analysing graft survival there was significant interaction between DCD transplants and biliary complications (*P* = 0·015), prompting subgroup analysis. On adjusted analysis the detrimental effects of biliary complications were exaggerated in DCD grafts (leak only aHR 1·290 (95 per cent c.i. 0·713 to 2·333), *P* = 0·401; stricture only aHR 1·733 (1·054 to 2·849), *P* = 0·030; leak plus stricture aHR 2·806 (1·587 to 4·961), *P* < 0·005) compared with donation after brain death (DBD) grafts (leak only aHR 1·349 (1·031 to 1·765), *P* = 0·029; stricture only aHR 1·459 (1·073 to 1·984), *P* = 0·016; leak plus stricture aHR 1·219 (0·702 to 2·119), *P* = 0·482).

A model without other early postoperative complications, but otherwise identical to the model in *Table 7*, was constructed. In this model the effect size of all of these biliary complications remained significant and was larger: leak aHR 1·790 (95 per cent c.i. 1·418 to 2·261), *P* < 0·001), stricture aHR 1·700 (1·318 to 2·192), *P* < 0·001) and leak plus stricture aHR 1·657 (1·120 to 2·451), *P* = 0·011).

Further sensitivity analyses were performed where the following factors were included in the model: MELD/UKELD, super-urgent listing, portal vein thrombus, aberrant hepatic artery anatomy. These factors did not have significant effects on the outcome of the analysis and were not significant in an adjusted model.

### Patient survival

**Fig. 2:**
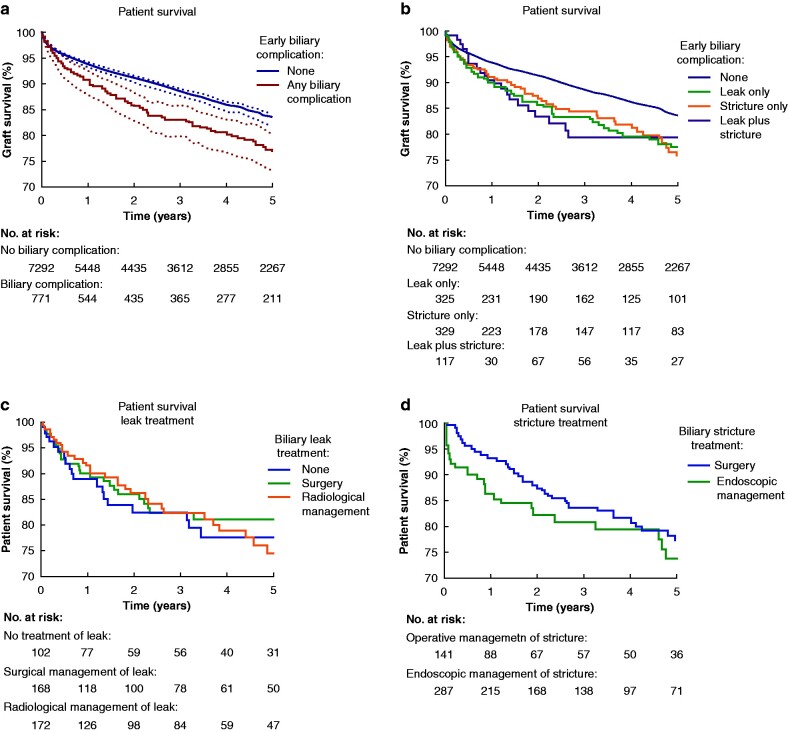
Impact of early anastomotic biliary complications on patient survival Each panel is a Kaplan–Meier graph, with numbers at risk displayed. *P* values are the result of log-rank tests. **a***P* < 0.001. Shaded areas represent 95 per cent confidence intervals. **b** *P* < 0.001. **c***P* = 0.637. **d** *P* = 0.155

Patient survival data was available for 8063 participants. *Figure 2* displays unadjusted analysis of patient survival in Kaplan–Meier plots. Patients with EABC had increased mortality (*Fig. 2a,b*). Overall, estimated 5-year patient survival was 83·3 (95 per cent c.i. 82·1 to 84·3) per cent in patients without an EABC, and 76·9 (72·9 to 80·4) per cent in the any biliary complication group; *P* < 0·001, (*Fig. 2a*). For both leak and stricture the chosen treatment modality did not significantly impact patient survival (*Fig. 2c,d*); these analyses on the effect of treatment modality are unadjusted, and large selection biases probably exist.

In the adjusted Cox regression model (*Table 8*) stricture (aHR 1·526 (95 per cent c.i. 1·183 to 1·968), *P* = 0·001) and leak plus stricture (aHR 1·509 (1·013 to 2·248), *P* = 0·043) were both independent predictors of increased patient mortality. Leak only was a significant predictor of patient survival on univariable analysis (HR 1·406 (95 per cent c.i. 1·098–1·799), *P* = 0·007), but significance was lost on adjusted Cox regression (aHR 1·215 (95 per cent c.i. 0·941 to 1·568), *P* = 0·136).

**Table 8:** Patient survival Cox regression using pooled data from all 20 imputed datasets (*n* = 8063 per imputation)

	Univariable		Adjusted	
Variable	Hazard ratio	P	Hazard ratio	P
**Biliary complication**				
None	1		1	
Leak only	1·406 (1·098–1·799)	0·007	1·215 (0·941–1·568)	0·136
Stricture only	1·523 (1·186–1·957)	0·001	1·526 (1·183–1·968)	0·001
Leak plus stricture	1·579 (1·062–2·347)	0·024	1·509 (1·013–2·248)	0·043
**Indication**				
Non-biliary	1		1	
Primary biliary cirrhosis	0·773 (0·621–0·962)	0·021	0·754 (0·604–0·940)	0·012
Primary sclerosing cholangitis	0·900 (0·733–1·104)	0·310	0·988 (0·804–1·215)	0·910
Biliary atresia	0·789 (0·296–2·107)	0·637	1·473 (0·582–3·728)	0·443
**Recipient age**	1·021 (1·015–1·026)	<0·001	1·025 (1·019–1·030)	<0·001
**Transplant year**	0·957 (0·938–0·977)	<0·001	0·937 (0·918–0·957)	<0·001
**Previous non-liver transplants**				
0	1		1	
1	2·737 (1·366–5·487)	0·005	1·592 (0·765–3·312)	0·214
2	5·348 (0·752–38·014)	0·094	10·094 (3·702–27·520)	0·021
**Previous liver transplants**				
0	1		1	
1	0·603 (0·453–0·802)	0·001	0·520 (0·386–0·700)	<0·001
2	0·941 (0·470–1·887)	0·865	1·176 (0·603–2·294)	0·650
>2	3·723 (1·395–9·938)	0·009	6·195 (2·918–13·154)	<0·001
**Donor age**	1·010 (1·007–1·014)	<0·001	1·011 (1·007–1·014)	<0·001
**Postoperative RRT**				
Nil	1		1	
Transient filtration	1·820 (1·558–2·127)	<0·001	1·914 (1·623–2·256)	<0·001
Short-term dialysis	2·225 (1·877–2·636)	<0·001	2·183 (1·823–2·614)	<0·001
Long-term dialysis	8·860 (5·243–14·973)	<0·001	7·518 (4·550–12·422)	<0·001
**HA thrombus**	2·439 (2·171–2·739)	<0·001	2·427 (1·924–3·062)	<0·001
**Fungal infection**	2·113 (1·569–2·846)	<0·001	1·654 (1·214–2·254)	0·001
**Sepsis**	1·380 (1·232–1·544)	<0·001	1·152 (1·021–1·300)	0·022

Values in parentheses are 95 per cent confidence intervals. These variables were selected using a backwards stepwise approach. RRT, renal replacement therapy; HA, hepatic artery.

Again, the authors re-analysed the data with the exclusion of other early postoperative complications. Interestingly, in this analysis the effect of leak only on patient survival reached statistical significance (leak aHR 1·506 (95 per cent c.i. 1·327 to 1·709), *P* = 0·001). The effect size of other groups was also exaggerated: stricture aHR 1·626 (95 per cent c.i. 1·430 to 1·848), *P* < 0·001) and leak plus stricture aHR 1·610 (1·315 to 1·972), *P* = 0·018).

In contrast to the analysis on graft survival, there was no significant interaction between DCD *versus* DBD and biliary complications when analysing patient survival; the detrimental effects of biliary stricture on patient survival were similar across these groups. Sensitivity analyses were performed as described for graft survival, and the main results remained unchanged.

### Predictors of biliary complications

Predictors of EABCs in whole deceased donor liver transplantation (excluding split and reduced grafts) were identified using multiple logistic regression and are displayed in *Table 9*. Several risk factors were identified for biliary leak. DCD grafts, grafts with aberrant arterial anatomy and grafts from female donors all had higher risk of leak. Recipients with higher MELD and Asian recipients also had higher chance of leak. UKELD was not a retained predictive factor. Recipients who had had three or more previous liver transplants (12 patients) had a higher chance of leak. There was no evidence that patients with one to two previous liver transplants had a higher risk of leak.

**Table 9:** Predictors of biliary leak and stricture in whole deceased donor liver transplants, using pooled data from all 20 imputed datasets (*n* = 7555)

	Simple logistic regression		Adjusted	
	Odds ratio	P	**Odds ratio**	P
**Biliary leak risk factors**				
Recipient MELD	1·02 (1·00–1·03)	0·019	1·02 (1·00–1·03)	0·023
Vascular anastomosis time (per minute)	1·01 (1·00–1·02)	0·001	1·01 (1·00–1·02)	0·001
DCD graft (DBD as comparator)	1·19 (0·93–1·52)	0·168	1·42 (1·10–1·84)	0·007
Aberrant hepatic artery anatomy	1·37 (1·05–1·78)	0·020	1·37 (1·05–1·79)	0·021
Female donor	1·22 (0·99–1·51)	0·067	1·24 (1·00–1·54)	0·047
Biliary anastomosis				
CDCD	1		1	
Roux	1·28 (0·96–1·70)	0·091	1·15 (0·84–1·58)	0·394
T-tube	1·99 (1·21–3·28)	0·007	2·06 (1·23–3·42)	0·006
Stent	1·57 (0·37–6·63)	0·536	1·54 (0·36–6·53)	0·561
Previous liver transplants				
0	1		1	
1	1·34 (0·94–1·92)	0·104	1·21 (0·82–1·79)	0·347
2	1·26 (0·51–3·12)	0·624	1·12 (0·44–2·88)	0·810
≥3	6·95 (1·87–25·79)	0·004	6·07 (1·56–23·69)	0·009
Recipient ethnicity				
White	1		1	
Asian	0·64 (0·40–1·02)	0·058	0·61 (0·38–0·97)	0·038
Black	0·91 (0·48–1·74)	0·783	0·85 (0·44–1·63)	0·622
Other	0·60 (0·24–1·46)	0·256	0·58 (0·24–1·43)	0·236
**Biliary stricture risk factors**				
DCD graft (DBD as comparator)	1·37 (1·09–1·71)	0·007	1·31 (1·04–1·64)	0·022
Biliary anastomosis				
CDCD	1		1	
Roux	0·54 (0·38–0·77)	0·001	0·56 (0·39–0·80)	0·001
T-tube or stent	0·71 (0·37–1·36)	0·305	0·75 (0·39–1·44)	0·390

Values in parentheses are 95 per cent confidence intervals. Odds ratios from simple and multiple binary logistic regression are given. These variables were selected using a backwards stepwise approach. MELD, model for end-stage liver disease; DCD, donation following circulatory death; DBD, donation following brainstem death; CDCD, choledocho-choledochostomy; Roux, Roux-en-Y hepaticojejunostomy.

Two ‘intraoperative’ factors were predictors of anastomotic leak. Patients where a T-tube was used in the biliary anastomosis were more likely to suffer a leak when compared with standard CDCD anastomosis. An increasing vascular anastomosis time (also termed reperfusion time) was also associated with a higher risk of biliary leak; every 10-min addition increased the risk of leak by approximately 10·5 per cent. Overall, the effect of stents on either leak or stricture is uncertain, due to the small number of stents used in whole deceased donor grafts (24 patients).

Fewer risk factors for biliary stricture could be identified. Again, DCD grafts had higher risk of biliary stricture when compared with DBD grafts (aOR 1·31 (95 per cent c.i. 1·04 to 1·64), *P* = 0·022). Roux anastomosis was associated with decreased risk of stricture when compared with standard CDCD anastomosis (aOR 0·56 (95 per cent c.i. 0·39 to 0·80), *P* = 0·001).

The authors also performed logistic regression models on the entire cohort, including split and reduced grafts. These models returned similar results to above, with the addition of split grafts as a significant predictor of leak (aOR 2·92 (95 per cent c.i. 2·21 to 3·87), *P* < 0·001), and reduced grafts as a risk factor for both leak (aOR 4·69 (95 per cent c.i. 3·02 to 7·29), *P* < 0·001) and stricture (aOR 3·01 (1·83 to 4·95), *P* < 0·001) when compared with whole grafts.

Despite identifying significant risk factors, the r^2^ of both of these models was low (0·024 and 0·008 for leak and stricture models respectively), indicating that these complications are difficult to predict using information available to transplanting surgeons. Building a clinically useful scoring system was not possible using the variables collected in the NHSBT registry.

## Discussion

In this manuscript the authors present the first national registry analysis investigating the clinical impact and risk factors for EABCs. Prior to this study, there were no large multicentre analyses to provide clinically relevant data to transplant clinicians. EABCs are common after liver transplantation (9·6 per cent), and associated with increased duration of hospital stay and increased readmissions to hospital in the first year. The present study shows for the first time that the detrimental impacts of EABC on graft survival and mortality remain significant when adjusted for a wide range of donor, graft, recipient, operative and other early postoperative complications. Risk factors for EABC, including DCD grafts, grafts with aberrant arterial anatomy, choice of biliary anastomosis, vascular anastomosis time and recipient MELD, were identified. Many of these risk factors are related to increased ischaemia, either before, during or after transplantation (DCD, increased vascular anastomosis time and aberrant arterial anatomy respectively).

NHSBT does not record exactly how these EABCs were diagnosed. However, routine or protocol magnetic resonance cholangiopancreatography (MRCP) to look for subclinical complications is not performed in the majority of transplant units in the UK. The incidence of EABC described in this study is similar to that described in previous series^7–9^ indicating that the present analysis is based on the incidence of clinically significant EABCs, rather than EABCs picked up on imaging alone. Two findings support this. First, the rate of anastomotic stricture in this study is far lower than the anastomotic stricture rate when routine MRCP is performed after transplantation, an incidence which exceeds 40 per cent^24^. Second, 96 per cent of the patients recorded as anastomotic stricture in this study required treatment.

Several risk factors were identified for EABC. DCD grafts were the only common risk factor for both leak and stricture. Additional risk factors for leak included grafts with aberrant arterial anatomy, increasing recipient MELD, the use of a T-tube for biliary anastomosis, and increasing vascular anastomosis time in the recipient. The use of a Roux-en-Y anastomosis appeared to decrease stricture risk. However, although the authors could identify significant risk factors, the r^2^ of these models was low, and it was not possible to predict accurately whether an individual patient would develop an EABC. This suggests factors not coded in the NHSBT registry may play a key role, such as damage to biliary epithelium caused during retrieval and preservation, mismatch in bile duct diameter, suturing technique used and type of CDCD anastomosis^25^. It also suggests that technical error, which cannot be coded in the database, may be a factor in EABC occurrence^8^.

Previous smaller studies have suggested that EABCs are associated with morbidity and increased mortality^7^^,^^15–17^. Performing analyses using a large registry has allowed the present study to expand on this work and to quantify the negative effects, whilst adjusting for multiple confounders. It has shown for the first time that EABCs are independent predictors of graft loss and mortality, even when adjusting for a large range of donor and recipient factors.

One strength of the present study is the inclusion of postoperative complications as confounders. As EABCs are associated with other early postoperative complications, it could be argued that the detrimental effects seen in previous studies were simply explained by EABC being markers of the other complications which are known to be detrimental (such as acute rejection). For the first time, this study has shown that the effects of EABCs on graft and patient survival are independent of other postoperative complications.

In some situations, EABCs will probably be the cause of other complications, such as sepsis. Therefore, the model adjusted for these postoperative complications and may underestimate the effect size of EABCs. To address this limitation, the authors performed sensitivity analyses without other early complications; in these models both leak and stricture (alone or in combination) had a significant impact on both graft loss and mortality, and the effect size estimates were larger.

Further limitations include the selection bias that is inherent to any registry analysis. Missing data have the potential to introduce bias in any registry; the authors adopted well accepted techniques to deal with missing data^26^^,^^27^. Finally, the study was limited by the granularity of the registry. Some factors that the authors wanted to investigate were not recorded in the database and therefore could not be analysed, such as bile duct size, exact arterial anatomy, smoking after transplant and occurrence of non-anastomotic ischaemic-type biliary lesions.

This study has several implications for current clinical practice and future research. The biliary anastomosis has long been considered the least difficult of the liver transplant anastomoses^28^. In many units there is a tendency for this either to be left to junior or less experienced surgeons, or be completed by a less focused senior surgeon, who is fatigued after performing the vascular anastomoses. This could explain the seemingly paradoxical finding that complications at the biliary anastomosis are more common than complications at the vascular anastomoses, which are considered more technically challenging^7^. The authors hope this study will cause a shift in mind-set, prompting surgeons to give the same care and attention to the biliary anastomosis as they do to the vascular anastomoses.

Future research should focus on methods to prevent biliary complications, especially in the high-risk groups that this study has identified. In several specialties enhanced recovery after surgery (ERAS) protocols are common place^29^^,^^30^, and in the setting of liver transplantation small studies have suggested that ERAS is feasible and that early enteral nutrition reduces rates of EABC^31^^,^^32^. Further studies looking to optimize ERAS protocols in liver transplantation and investigating the effect on EABC occurrence would be valuable. In addition, further studies looking at radiological screening of high-risk groups and different management options are required.

As DCD grafts are at increased risk of both leak and stricture, and there are increasing reports of extensive biliary epithelial damage following retrieval and storage, improving preservation and optimizing organs is another potential avenue for EABC prevention^25^^,^^33^. The use of dynamic machine-perfusion techniques for organ preservation and optimization is seeing growing international interest. However, a systematic review described no evidence that any commonly used machine-perfusion technique was able to reduce EABC rate, which was also demonstrated in a recent randomized trial of normothermic machine perfusion^24^^,^^34^. It has been reported that hypothermic oxygenated perfusion is able to ameliorate ischaemic-type biliary lesions, suggesting it is able to prevent damage to biliary epithelium^33^. Randomized trials of hypothermic oxygenated perfusion are ongoing, and will add key evidence as to whether this dynamic-preservation modality is able to reduce the rate of EABCs^35^.

## Supplementary Material

zrab019_Supplementary_Data

## Notes

This manuscript was presented in the Patey Prize Session of Society of Research Surgery Virtual Meeting 2020 and nominated for the Calne Williams Medal at the British Transplant Society Congress 2020

## References

[1] NHS Blood and Transplant. *UK Annual Report on* L*iver Transplantation.* 2019. https://www.odt.nhs.uk/statistics-and-reports/organ-specific-reports/ (accessed 1 December 2020)

[2] Renz JF , Kin C, Kinkhabwala M, Jan D, Varadarajan R, Goldstein M et al Utilization of extended donor criteria liver allografts maximizes donor use and patient access to liver transplantation. Ann Surg 2005;242:556–563 16192816 10.1097/01.sla.0000183973.49899.b1 PMC1402340

[3] Monbaliu D , Pirenne J, Talbot D. Liver transplantation using donation after cardiac death donors. J Hepatol 2012;56:474–485 21782762 10.1016/j.jhep.2011.07.004

[4] Nemes B , Gámán G, Polak WG, Gelley F, Hara T, Ono S et al Extended-criteria donors in liver transplantation Part II: reviewing the impact of extended-criteria donors on the complications and outcomes of liver transplantation. Expert Rev Gastroenterol Hepatol 2016;10:841–859 26831547 10.1586/17474124.2016.1149062

[5] Briceño J , Ciria R, Pleguezuelo M, de la Mata M, Muntané J, Naranjo Á et al Impact of donor graft steatosis on overall outcome and viral recurrence after liver transplantation for hepatitis C virus cirrhosis. Liver Transpl 2009;15:37–48 19109846 10.1002/lt.21566

[6] Laing RW , Scalera I, Isaac J, Mergental H, Mirza DF, Hodson J et al Liver transplantation using grafts from donors after circulatory death: a propensity score-matched study from a single center. Am J Transplant 2016;16:1795–1804 26725645 10.1111/ajt.13699

[7] Kochhar G , Parungao JM, Hanouneh IA, Parsi MA. Biliary complications following liver transplantation. World J Gastroenterol 2013;19:2841–2846 23704818 10.3748/wjg.v19.i19.2841 PMC3660810

[8] Greif F , Bronsther OL, Van Thiel DH, Casavilla A, Iwatsuki S, Tzakis A et al The incidence, timing, and management of biliary tract complications after orthotopic liver transplantation. Ann Surg 1994;219:40–45 8297175 10.1097/00000658-199401000-00007 PMC1243088

[9] Laurence JM , Sapisochin G, DeAngelis M, Seal JB, Miserachs MM, Marquez M et al Biliary complications in pediatric liver transplantation: incidence and management over a decade. Liver Transpl 2015;21:1082–1090 25991054 10.1002/lt.24180

[10] Rela M , Vougas V, Muiesan P, Vilca-Melendez H, Smyrniotis V, Gibbs P et al Split liver transplantation: King's College Hospital experience. Ann Surg 1998;227:282–288 9488528 10.1097/00000658-199802000-00019 PMC1191247

[11] Theilmann L , Küppers B, Kadmon M, Roeren T, Notheisen H, Stiehl A et al Biliary tract strictures after orthotopic liver transplantation: diagnosis and management. Endoscopy 1994;26:517–522 7828563 10.1055/s-2007-1009026

[12] Suárez F , Otero A, Solla M, Arnal F, Lorenzo MJ, Marini M et al Biliary complications after liver transplantation from Maastricht category-2 non-heart-beating donors. Transplantation 2008;85:9–14 18192905 10.1097/01.tp.0000297945.83430.ce

[13] Halme L , Hockerstedt K, Lautenschlager I. Cytomegalovirus infection and development of biliary complications after liver transplantation. Transplantation 2003;75:1853–1858 12811245 10.1097/01.TP.0000064620.08328.E5

[14] Kienlein S , Schoening W, Andert A, Kroy D, Neumann UP, Schmeding M. Biliary complications in liver transplantation: impact of anastomotic technique and ischemic time on short- and long-term outcome. World J Transplant 2015;5:300–309 26722658 10.5500/wjt.v5.i4.300 PMC4689941

[15] Foley DP , Fernandez LA, Leverson G, Anderson M, Mezrich J, Sollinger HW et al Biliary complications after liver transplantation from donation after cardiac death donors: an analysis of risk factors and long-term outcomes from a single center. Ann Surg 2011;253:817–825 21475025 10.1097/SLA.0b013e3182104784 PMC3103075

[16] Rammohan A , Govil S, Vargese J, Kota V, Reddy MS, Rela M. Changing pattern of biliary complications in an evolving liver transplant unit. Liver Transpl 2017;23:478–486 28152569 10.1002/lt.24736

[17] Thethy S , Thomson BN, Pleass H, Wigmore SJ, Madhavan K, Akyol M et al Management of biliary tract complications after orthotopic liver transplantation. Clin Transplant 2004;18:647–653 15516238 10.1111/j.1399-0012.2004.00254.x

[18] Axelrod DA , Dzebisashvilli N, Lentine KL et al National assessment of early biliary complications after liver transplantation: economic implications. Transplantation 2014;98:1226–1235 25119126 10.1097/TP.0000000000000197

[19] Ostroff JW. Management of biliary complications in the liver transplant patient. Gastroenterol Hepatol (N Y) 2010;6:264–272 20567581 PMC2886474

[20] Shin M , Joh JW. Advances in endoscopic management of biliary complications after living donor liver transplantation: comprehensive review of the literature. World J Gastroenterol 2016;22:6173–6191 27468208 10.3748/wjg.v22.i27.6173 PMC4945977

[21] Memeo R , Piardi T, Sangiuolo F, Sommacale D, Pessaux P. Management of biliary complications after liver transplantation. World J Hepatol 2015;7:2890–2895 26689137 10.4254/wjh.v7.i29.2890 PMC4678375

[22] Collett D , Friend PJ, Watson CJ. Factors associated with short- and long-term liver graft survival in the United Kingdom: development of a UK Donor Liver Index. Transplantation 2017;101:786–792 27906826 10.1097/TP.0000000000001576 PMC7228599

[23] Asrani SK , Saracino G, O'Leary JG, Gonzales S, Kim PT, McKenna GJ et al Recipient characteristics and morbidity and mortality after liver transplantation. J Hepatol 2018;69:43–50 29454069 10.1016/j.jhep.2018.02.004

[24] Nasralla D , Coussios CC, Mergental H, Akhtar MZ, Butler AJ, Ceresa CDL et al; Consortium for Organ Preservation in Europe. A randomized trial of normothermic preservation in liver transplantation. Nature 2018;557:50–56 29670285 10.1038/s41586-018-0047-9

[25] de Jong IEM , Matton APM, van Praagh JB, van Haaften WT, Wiersema‐Buist J, van Wijk LA et al Peribiliary glands are key in regeneration of the human biliary epithelium after severe bile duct injury. Hepatology (Baltimore, Md.) 2019;69:1719–1734 10.1002/hep.30365 PMC6594148 30506902

[26] Schlegel A , Kalisvaart M, Scalera I, Laing RW, Mergental H, Mirza DF et al The UK DCD Risk Score: a new proposal to define futility in donation-after-circulatory-death liver transplantation. J Hepatol 2018;68:456–464 29155020 10.1016/j.jhep.2017.10.034

[27] Sterne JAC , White IR, Carlin JB, Spratt M, Royston P, Kenward MG et al Multiple imputation for missing data in epidemiological and clinical research: potential and pitfalls. BMJ 2009;338:b2393 19564179 10.1136/bmj.b2393 PMC2714692

[28] Klein AS , Savader S, Burdick JF, Fair J, Mitchell M, Colombani P et al Reduction of morbidity and mortality from biliary complications after liver transplantation. Hepatology (Baltimore, Md) 1991;14:818–823 10.1002/hep.1840140513 1937387

[29] Gustafsson UO , Scott MJ, Hubner M, Nygren J, Demartines N, Francis N et al Guidelines for perioperative care in elective colorectal surgery: Enhanced Recovery After Surgery (ERAS^®^) Society recommendations: 2018. World J Surg 2019;43:659–695 30426190 10.1007/s00268-018-4844-y

[30] Low DE , Allum W, De Manzoni G, Ferri L, Immanuel A, Kuppusamy M et al Guidelines for perioperative care in esophagectomy: Enhanced Recovery After Surgery (ERAS^®^) Society Recommendations. World J Surg 2019;43:299–330 30276441 10.1007/s00268-018-4786-4

[31] Brustia R , Monsel A, Conti F, Savier E, Rousseau G, Perdigao F et al Enhanced recovery in liver transplantation: a feasibility study. World J Surg 2019;43:230–241 30094639 10.1007/s00268-018-4747-y

[32] Kim JM , Joh J-W, Kim HJ, Kim S-H, Rha M, Sinn DH et al Early enteral feeding after living donor liver transplantation prevents infectious complications: a prospective pilot study. Medicine (Baltimore) 2015;94:e1771 26554774 10.1097/MD.0000000000001771 PMC4915875

[33] Op den Dries S , Westerkamp AC, Karimian N, Gouw ASH, Bruinsma BG, Markmann JF et al Injury to peribiliary glands and vascular plexus before liver transplantation predicts formation of non-anastomotic biliary strictures. J Hepatol 2014;60:1172–1179 24560661 10.1016/j.jhep.2014.02.010

[34] Boteon YL , Boteon AP, Attard J, Wallace L, Bhogal RH, Afford SC. Impact of machine perfusion of the liver on post-transplant biliary complications: a systematic review. World J Transplant 2018;8:220–231 30370232 10.5500/wjt.v8.i6.220 PMC6201326

[35] Czigany Z , Schöning W, Ulmer TF, Bednarsch J, Amygdalos I, Cramer T et al Hypothermic oxygenated machine perfusion (HOPE) for orthotopic liver transplantation of human liver allografts from extended criteria donors (ECD) in donation after brain death (DBD): a prospective multicentre randomised controlled trial (HOPE ECD-DBD). BMJ Open 2017;7:e017558 10.1136/bmjopen-2017-017558 PMC5652559 29018070

