# Supplementary material for: Risk factors and impact of early anastomotic biliary complications after liver transplantation: UK registry analysis
Source: BJS Open. 2021 Apr 15;5(2):zrab019. doi: 10.1093/bjsopen/zrab019 (PMC8047096; doi:10.1093/bjsopen/zrab019)
Supplement: zrab019_Supplementary_Data [file zrab019_supplementary_data.docx]

# Supplementary Content

Supplementary table 1 – Notes on data cleaning

| **Variable** | **Details of data cleaning** |
| --- | --- |
| Length of stay | 3 values removed as reported as >10000 |
| Cold ischaemic time | DCD/DBD with <30min CIT data removed (n=3), CIT of 9999 removed (n=70). |
| Total warm ischaemic time | Total WIT >1000 and <5 data deleted (n=7) |
| Functional warm ischaemic time | 1 value >1000 was removed |
| Reperfusion time | 9999 recoded to missing |
| 1 year creatinine | Values <15 or coded as 999 deleted (n=10) |
| Length of stay | Values 0,1,2,3 were deleted (n=28) |
| Donor bilirubin | Single value of 0 deleted |
| BMI | BMI data was deleted for 10 records (9 where BMI was <13 and one where BMI was 145 removed) |

Supplementary table 2 - breakdown of missing data

|  | Missing | | Valid N |
| --- | --- | --- | --- |
|  | N | Percent |  |
| Donor bilirubin | 2408 | 29·0% | 5896 |
| Recipient diabetes | 1927 | 23·2% | 6377 |
| Hepatic artery anastomosis (single versus multiple) | 1495 | 18·0% | 6809 |
| Hepatic artery anatomy | 1378 | 16·6% | 6926 |
| Vascular anastomosis time | 1338 | 16·1% | 6966 |
| Waiting list time | 1165 | 14·0% | 7139 |
| UKELD | 1130 | 13·6% | 7174 |
| MELD | 1127 | 13·6% | 7177 |
| Donor cardiovascular disease | 469 | 5·6% | 7835 |
| Donor hypertension | 432 | 5·2% | 7872 |
| Donor diabetes | 360 | 4·3% | 7944 |
| Donor smoker | 338 | 4·1% | 7966 |
| Steatosis | 253 | 3·0% | 8051 |
| Liver failure grade | 214 | 2·6% | 8090 |
| Recipient encephalopathy grade | 130 | 1·6% | 8174 |
| Renal replacement therapy post op | 97 | 1·2% | 8207 |
| Rejection episodes | 92 | 1·1% | 8212 |
| Cold ischaemic time | 86 | 1·0% | 8218 |
| Biliary anastomosis type | 74 | 0·9% | 8230 |
| Donor ethnicity | 69 | 0·8% | 8235 |
| Sepsis post op | 45 | 0·5% | 8259 |
| RRT before transplant | 30 | 0·4% | 8274 |
| Fungal infection post op | 26 | 0·3% | 8278 |
| CMV infection post op | 24 | 0·3% | 8280 |
| Donor sex | 3 | 0·0% | 8301 |
| Portal vein thrombus post op | 3 | 0·0% | 8301 |
| Hepatic artery thrombus post op | 2 | 0·0% | 8302 |
| Donor age | 1 | 0·0% | 8303 |
| Biliary complication post op | 0 | 0·0% | 8304 |
| Donor type (DCD, DBD, Living or domino) | 0 | 0·0% | 8304 |
| Previous liver transplants | 0 | 0·0% | 8304 |
| Previous total transplants | 0 | 0·0% | 8304 |
| Whole versus split versus reduced | 0 | 0·0% | 8304 |
| Super-urgent listing | 0 | 0·0% | 8304 |
| Transplant year | 0 | 0·0% | 8304 |
| Indication for transplant | 0 | 0·0% | 8304 |
| Recipient age | 0 | 0·0% | 8304 |

Supplementary Table 3 – Donor and recipient factors associated with early anastamotic biliary complications. Percentages displayed describe biliary complication rate for each factor (within row percentages). Continuous variables are displayed as mean ± standard deviation, unless stated otherwise. For categorical variables P-values are the result of Chi-Square tests, except those marked by ^a^, where Monte-Carlo simulations of Fishers exact tests were used. For continuous variables P-values are the result of one-way ANOVA, except those marked by ^b^ where Kruskall-Wallis was used. Raw data is given, analysis of missing data is found in supplementary table 2. CVS – cardiovascular system, BMI – body mass index, Total warm ischaemic time – time from withdrawal to aortic flush, ALT – alanine transaminase, ALP – alkaline phosphatase, GGT – gamma glutamyl transpeptidase, RRT – renal replacement therapy, UKELD – UK model for end-stage liver disease.

|  | No biliary complication (n=7505) | Biliary leak alone (n=344) | Biliary stricture alone (n=335) | Both leak and stricture (n=120) | P-value |
| --- | --- | --- | --- | --- | --- |
| **Donor blood parameters**, median (range) |  |  |  |  |  |
| ALT | 30 (3-4220), n=4808 | 30 (8-805), n=204 | 30 (8-3860), n=204 | 27·5 (7-401), n=78 | 0·526^b^ |
| ALP | 70 (2-1863), n=5291) | 67 (18-944), n=232 | 71 (18-441), n=217 | 74·5 (40-492), n=84 | 0·191^b^ |
| Bilirubin | 9 (1-108), n=5363 | 9 (2-44), n=230 | 9 (2-71), n=219 | 8 (3-54), n=84 | 0·948^b^ |
| GGT | 34 (3-1650), n=3808 | 32 (1-558), n=153 | 36 (6-1031), n=166 | 29 (7-257), n=54 | 0·422^b^ |
| **Previous liver transplants** |  |  |  |  |  |
| 0 | 6846 (90·4%) | 306 (4·0%) | 313 (4·1%) | 111 (1·5%) | 0·301^a^ |
| 1 | 569 (90·9%) | 32 (5·1%) | 19 (3·0%) | 6 (1·0%) |  |
| ≥1 | 90 (88·2%) | 6 (5·9%) | 3 (2·9%) | 3 (2·9%) |  |
| **Previous organ transplants** |  |  |  |  |  |
| 0 | 6824 (90·4%) | 305 (4·0%) | 312 (4·1%) | 111 (1·5%) | 0·279^a^ |
| 1 | 587 (91·1%) | 32 (5·0%) | 19 (3·0%) | 6 (0·9%) |  |
| ≥1 | 94 (87·0%) | 7 (6·5%) | 4 (3·7%) | 3 (2·8%) |  |
| **UKELD** | 54·8±5·7, n=6491 | 55·5±6·1, n=295 | 54·5±5·7, n=285 | 54·6±5·5, n=103 | 0·162 |
| **Median waiting list time in days (range)** | 86 (0-2425), n=6468 | 86 (0-1587), n=289 | 80 (0-1473), n=285 | 89 (0-1170), n=97 | 0·584^b^ |
| **Recipient ethnicity** |  |  |  |  |  |
| White | 6476 (90·5%) | 298 (4·2%) | 277 (3·9%) | 104 (1·5%) | 0·515 |
| Asian | 611 (90·1%) | 28 (4·1%) | 33 (4·9%) | 6 (0·9%) |  |
| Black | 218 (89·7%) | 8 (3·3%) | 13 (5·3%) | 4 (1·6%) |  |
| Other | 197 (87·6%) | 10 (4·4%) | 12 (5·3%) | 6 (2·7%) |  |
| **Recipient smoking** |  |  |  |  |  |
| Non-smoker | 3455 (90·3%) | 155 (4·1%) | 155 (4·1%) | 62 (1·6%) | 0·709 |
| Ex-smoker | 1523 (89·5%) | 79 (4·6%) | 74 (4·4%) | 25 (1·5%) |  |
| Current smoker | 731 (91·0%) | 25 (3·1%) | 34 (4·2%) | 13 (1·6%) |  |
| **Encephalopathy grade** |  |  |  |  |  |
| 0-1 | 6382 (90·4%) | 296 (4·2%) | 278 (3·9%) | 104 (1·5%) | 0·730 |
| 2-4 | 1007 (90·4%) | 43 (3·9%) | 50 (4·5%) | 14 (1·3%) |  |
| **Recipient past medical history** |  |  |  |  |  |
| Diabetic | 1384 (90·2%) | 56 (3·6%) | 70 (4·6%) | 25 (1·6%) | 0·595 |
| Non-diabetic | 4364 (90·1%) | 207 (4·3%) | 196 (4·0%) | 75 (1·5%) |  |
| RRT pre-transplant | 831 (91·3%) | 37 (4·1%) | 31 (3·4%) | 11 (1·2%) | 0·683 |
| No RRT | 6647 (90·3%) | 306 (4·2%) | 302 (4·1%) | 109 (1·5%) |  |
| Recipient BMI | 27·0±5·2, n=7447 | 26·8±5·4, n=341 | 27·2±5·4, n=331 | 27·4±5·2, n=118 | 0·700 |
| **Oesophageal varices** |  |  |  |  |  |
| None | 2999 (90·4%) | 141 (4·2%) | 122 (3·7%) | 56 (1·7%) | 0·122 |
| Present without previous bleed | 2804 (91·0%) | 109 (3·5%) | 129 (4·2%) | 41 (1·3%) |  |
| Previous variceal bleed | 1614 (89·6%) | 89 (4·9%) | 78 (4·3%) | 21 (1·2%) |  |
